# Gut Resistome of Preschool Children After Prolonged Mass Azithromycin Distribution: A Cluster-randomized Trial

**DOI:** 10.1093/cid/ciab485

**Published:** 2021-05-26

**Authors:** Ahmed M Arzika, Ramatou Maliki, Amza Abdou, Alio K Mankara, Abdoul N Harouna, Catherine Cook, Armin Hinterwirth, Lee Worden, Lina Zhong, Cindi Chen, Kevin Ruder, Zhaoxia Zhou, Elodie Lebas, Kieran S O’Brien, Catherine E Oldenburg, Victoria Le, Benjamin F Arnold, Travis C Porco, Jeremy D Keenan, Thomas M Lietman, Thuy Doan

**Affiliations:** 1 The Carter Center, Niamey, Niger; 2 Ministry of Health, Niamey, Niger; 3 Programme National de Santé Oculaire, Niamey, Niger; 4 Francis I Proctor Foundation, Universit of California San Francisco, San Francisco, California, USA; 5 Department of Ophthalmology, University of California San Francisco, San Francisco, California, USA; 6 Department of Epidemiology and Biostatistics, University of California San Francisco, San Francisco, California, USA; 7 Institute for Global Health Sciences, University of California San Francisco, San Francisco, California, USA

**Keywords:** azithromycin, antibiotic resistance, gut resistome, mass drug distribution, Niger, preschool children

## Abstract

We evaluated the gut resistome of children from communities treated with 10 twice-yearly azithromycin distributions. Although the macrolide resistance remained higher in the azithromycin arm, the selection of non-macrolide resistance observed at earlier time points did not persist. Longitudinal resistance monitoring should be a critical component of mass distribution programs.

**Clinical trials registration:**

NCT02047981

Periodic mass azithromycin administration to Nigerien children to reduce childhood mortality resulted in the selection of both macrolide and non-macrolide resistance determinants in the gut [1]. This coresistance, the acquisition of resistance genes affecting multiple antimicrobial classes, only became apparent after 6 biannual treatments (3 years) and persisted after 8 biannual treatments (4 years) [1]. The potential emergence of multidrug resistance in populations receiving repeated mass distribution of a single antibiotic agent questions the long-term usefulness of these programs. In this cluster-randomized controlled trial, we evaluated the gut resistome of children from those same communities after 5 years of biannual treatments.

## METHODS

### Trial Methods

The University of California, San Francisco (UCSF) Committee for Human Research and the Ethical Committee of the Niger Ministry of Health (IRB no. 10-01036) provided ethical oversight, and activities followed the Declaration of Helsinki. Due to Niger’s low literacy rate, we obtained verbal informed consent from guardians of children prior to treatment and swab collection [2]. No incentives were offered.

MORDOR (*Macrolides Oraux pour Réduire les Décès avec un Oeil sur la Résistance*) (NCT02047981) was a cluster-randomized trial that evaluated the effect of biannual azithromycin treatment on childhood mortality in Niger, Malawi, and Tanzania [3]. In Niger’s Dosso region where the mortality trial was being conducted, 30 communities were randomly selected for enrollment in a sister trial to monitor resistance outcomes. Communities were randomized 1:1 to receive either azithromycin or placebo. All children aged 1–59 months and weighing ≥3800 grams were eligible for treatment. Treatment was either a single oral dose of placebo or azithromycin (weight-based dosing for those who could not stand and height-based dosing for those who could to a target dose of ≥20 mg/kg) approximately every 6 months for 5 years. All field and laboratory personnel were masked to the assignments.

A random sample of 40 children per community was selected for rectal sample collection at annual monitoring visits (Supplementary Figure 1). Repeated cross-sectional samples were taken at each visit, so children may have been sampled repeatedly. Rectal swabs were obtained at baseline (prior to treatment), 24 months (6 months after the 4th treatment), 36 months (6 months after the 6th treatment), 48 months (6 months after the 8th treatment, and 60 months (6 months after the 10th treatment). Samples were placed in the Norgen stool collection kit to preserve nucleic acid integrity, and placed on ice packs in the field, stored at −20°C in Niger, and shipped to UCSF at 4ºC for long-term storage at −80°C until sample processing. Sample collection for the 60-month time point occurred from 4 February 2020 to 22 March 2020. Gut resistome results for all time points except for the 60 months have been published [1, 3, 4].

### Laboratory Methods

Samples from 29 villages were assessed. All rectal samples collected were pooled at the village level for metagenomic DNA sequencing to evaluate gut antibiotic resistance determinants (Supplementary Figure 1 and Supplementary Table 1) [3]. Nucleic acid extraction and sequencing libraries were prepared and sequenced as previously described [4]. Briefly, total DNA was extracted from the pooled rectal samples using the Norgen stool DNA isolation kit (Norgen) per manufacturer’s instructions. The extracted pooled DNA was used to prepare DNA sequencing libraries using the New England Biolabs’ (NEB) NEBNext Ultra II DNA Library Prep Kit and then amplified with 10 PCR cycles and sequenced on the NovaSeq 6000 instrument using 150-nucleotide (nt) paired-end sequencing.

### Statistical Methods

Sequencing data were analyzed for antibiotic resistance determinants as previously described [1]. Briefly, host reads were removed, and the remaining non-host reads were aligned to the MEGARes reference antimicrobial database (version 1.0.1) [5]. We estimated >80% power to detect a 16% difference (comparing 12%–28%) of resistance determinants in the gut. The primary outcome was the normalized abundance of macrolide resistance determinants at the class level between arms. Secondary outcomes were the resistance determinants of other antimicrobial classes, including a difference in the normalized abundance of betalactams reads. A Benjamini-Hochberg correction was used, allowing for a 5% false discovery rate.

## RESULTS

Rectal swabs were collected approximately 6 months after the 10th treatment (60-month time point) and analyzed from 1,004 children (557 from 15 placebo-treated communities and 447 from 14 azithromycin-treated communities). One community declined further participation after 24 months. Approximately half of the children were female and the median age was 32 months (Supplementary Table 1). Treatment data were missing for one of the villages in the azithromycin arm at the 54-month time point (Supplementary Figure 1). The mean (standard deviation) treatment coverage was 87.7% (11.9%) for the placebo arm and 84.5% (16.2%) for the azithromycin arm over the 5-year study duration.

**Figure 1.**
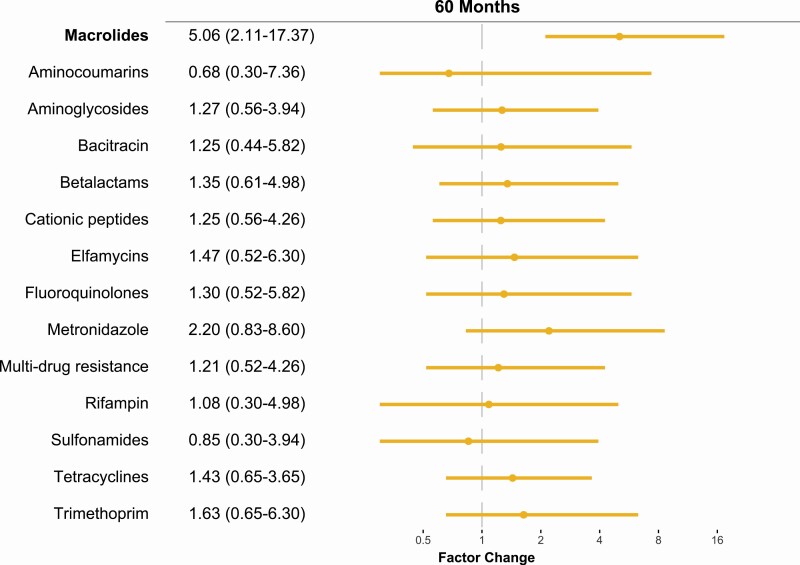
Gut antimicrobial resistance determinants of children 6 months after the 10th twice-yearly oral azithromycin distribution. Factor difference of antibiotic resistance determinants in the azithromycin treated group compared to the placebo treated group with associated 95% confidence interval (95% CI).

At 60 months, there was a 5.1-factor increase (95% confidence interval 2.1 to 17.4-factor change, *P* = .01, Figure 1) in the normalized abundance of macrolide resistance determinants in communities treated with azithromycin compared to communities treated with placebo. The additional 2 rounds of mass azithromycin distributions did not lead to a higher overall increase in macrolide resistance determinants compared to the 48-month time point (Supplementary Figure 2). In contrast to the results at 36- and 48-month time points, no significant differences were seen between treatment arms for other antibiotic classes, including betalactam resistance determinants (Figure 1 and Supplementary Figure 3).

## DISCUSSION

We evaluated the gut resistome of children from communities that received biannual azithromycin distributions for 5 years. Although the selection for macrolide resistance persisted, the overall change in resistance determinants did not increase with an additional 2 rounds of azithromycin distributions (7.4-factor higher (95% confidence interval [CI]: 4.0–16.7) at 36 months and 7.5-factor higher (95% CI: 3.8–23.1) at 48 months) [1]. Furthermore, we were unable to detect a significant difference in non-macrolide resistance determinants between arms. These results are in contrast to findings at earlier time points in the same trial, where resistance determinants for non-macrolide classes of antibiotics were selected for in azithromycin communities [1].

The MORDOR trial in Niger started in 2014 [6]. The area was chosen for its high childhood mortality rate, rural location, and lack of azithromycin distribution for trachoma over the prior 5 years. Of the 646 communities in the Dosso region that were eligible for the mortality study, 30 of those communities were randomized to this study to closely monitor the effects of azithromycin. These 30 communities were surrounded by communities in the larger MORDOR trial. Although the treatment assignment of the 30 communities did not change over the 5-year study, the treatment assignment of the surrounding communities did, with all communities receiving azithromycin the third year [6, 7]. Therefore, by 36 months, only the 15 communities in the present study remained azithromycin-naïve out of the enrollment area. In this setting, physical contact between participants of neighboring villages could have resulted in the exchange of microbes and any associated antimicrobial resistance (ie, contamination) [8, 9]. Indeed, the pattern of macrolide resistance in this study mirrors the amount of antibiotic selection pressure in the surrounding communities of the larger trial, with the highest abundance of macrolide resistance seen at the month 36 study visit, which corresponds to the 1-year time period when virtually all surrounding communities were receiving azithromycin (Supplementary Figure 2) [10]. It has been demonstrated that as little as 1% contact between populations resulted in a reduction of 30–50% of the expected change in antimicrobial resistance [10]. We therefore cannot rule out broader geographic spillover as a contributing factor for the lack of observed differences in antibiotic resistance between arms.

The acquisition of an antibiotic resistance gene can be associated with a fitness cost to the organism, and this effect can be cumulative with the addition of other resistance genes [11, 12]. In such a scenario, additional mass drug distributions may not necessarily result in the worsening of antimicrobial resistance as the system could eventually reach equilibrium. Although we cannot rule out the effect of spillover, the selection of macrolide resistance determinants under the pressure of mass drug distribution appeared to have saturated after the 6th distribution. The distribution of the normalized abundance of macrolide resistance genetic determinants appeared to be bimodal at 60 months. Approximately one third of azithromycin-treated communities had a high abundance, and the remaining had an abundance in the range of the placebo-treated communities. This may represent a different population or may be a statistical anomaly, given that the distribution was not bimodal at previous time points.

This cluster-randomized trial is limited by the absence of phenotypic resistance and the lack of data on the daily movements of individuals between communities. Another limitation is the lack of prevalence data on antimicrobial resistance determinants, which cannot be determined when samples are pooled. We were also unable to take into account antibiotic use outside of the study. Finally, the study was not designed to assess clinical outcomes such as pneumonia or diarrhea.

In summary, we did not detect a difference in non-macrolide resistance determinants in the stool of children from communities that received biannual azithromcyin distributions for 5 years compared to those that had received biannual placebo. Masked, randomized treatment allocation can eliminate many biases, but broader geographic spillover effects from activities beyond the study population may limit comparison in the long term.

## Supplementary Data

Supplementary materials are available at *Clinical Infectious Diseases* online. Consisting of data provided by the authors to benefit the reader, the posted materials are not copyedited and are the sole responsibility of the authors, so questions or comments should be addressed to the corresponding author.

ciab485_suppl_Supplementary_Materials

## Notes

***Funding.*** This work was funded by the Bill and Melinda Gates Foundation, the National Eye Institute of the National Institutes of Health (NIH) under award number K08EY026986, the Peierls Foundation, Research to Prevent Blindness Career Development Award, and an unrestricted grant from Research to Prevent Blindness (RPB, New York, NY, USA). Pfizer donated all medications used in this study.

***Potential conflicts of interest.*** T. D. and C. O. report grants from Bill and Melinda Gates Foundation outside the submitted work. B. A. reports grants from NIH/National Institute of Allergy and Infectious Diseases and Task Force for Global Health outside the submitted work; advisory board participation from NIH/National Eye Institute; travel fees from Bill and Melinda Gates Foundation outside the submitted work. T. L. reports grants from Bill and Melinda Gates Foundation outside the submitted work. T. C. P. served on the Data and Safety Monitoring Board (DSMB) for an azithromycin trial conducted by J. Walson, as well as on a DSMB for the SANTE trial in Mali (K. Kotloff, principal investigator [PI]) outside the submitted work. All other authors report no potential conflicts. All authors have submitted the ICMJE Form for Disclosure of Potential Conflicts of Interest. Conflicts that the editors consider relevant to the content of the manuscript have been disclosed.
