# Supplementary material for: Gut Resistome of Preschool Children After Prolonged Mass Azithromycin Distribution: A Cluster-randomized Trial
Source: Clin Infect Dis. 2021 May 26;73(7):1292–5. doi: 10.1093/cid/ciab485 (PMC8492121; doi:10.1093/cid/ciab485)
Supplement: ciab485_suppl_Supplementary_Materials [file ciab485_suppl_supplementary_materials.pdf]

Supplementary Materials for

Gut resistome of preschool children after prolonged mass azithromycin distribution: a cluster-randomized trial.

Ahmed M. Arzika[1], Ramatou Maliki[1], Amza Abdou[2,3], Alio K. Mankara[1], Abdoul N. Harouna[1], Catherine Cook[4], Armin Hinterwirth[4], Lee Worden[4], Lina Zhong[4], Cindi Chen[4], Kevin Ruder[4], Zhaoxia Zhou[4], Elodie Lebas[4], Kieran S. O'Brien[4], Catherine E. Oldenburg[4,5,6], Victoria Le[4], Benjamin Arnold[4], Travis Porco[4,5,6], Jeremy D. Keenan[4,5,6], Thomas M. Lietman[4,5,6,7], Thuy Doan[4,5]

This PDF file includes:

Figures S1-S3

Tables S1

Figure S1. Trial Profile

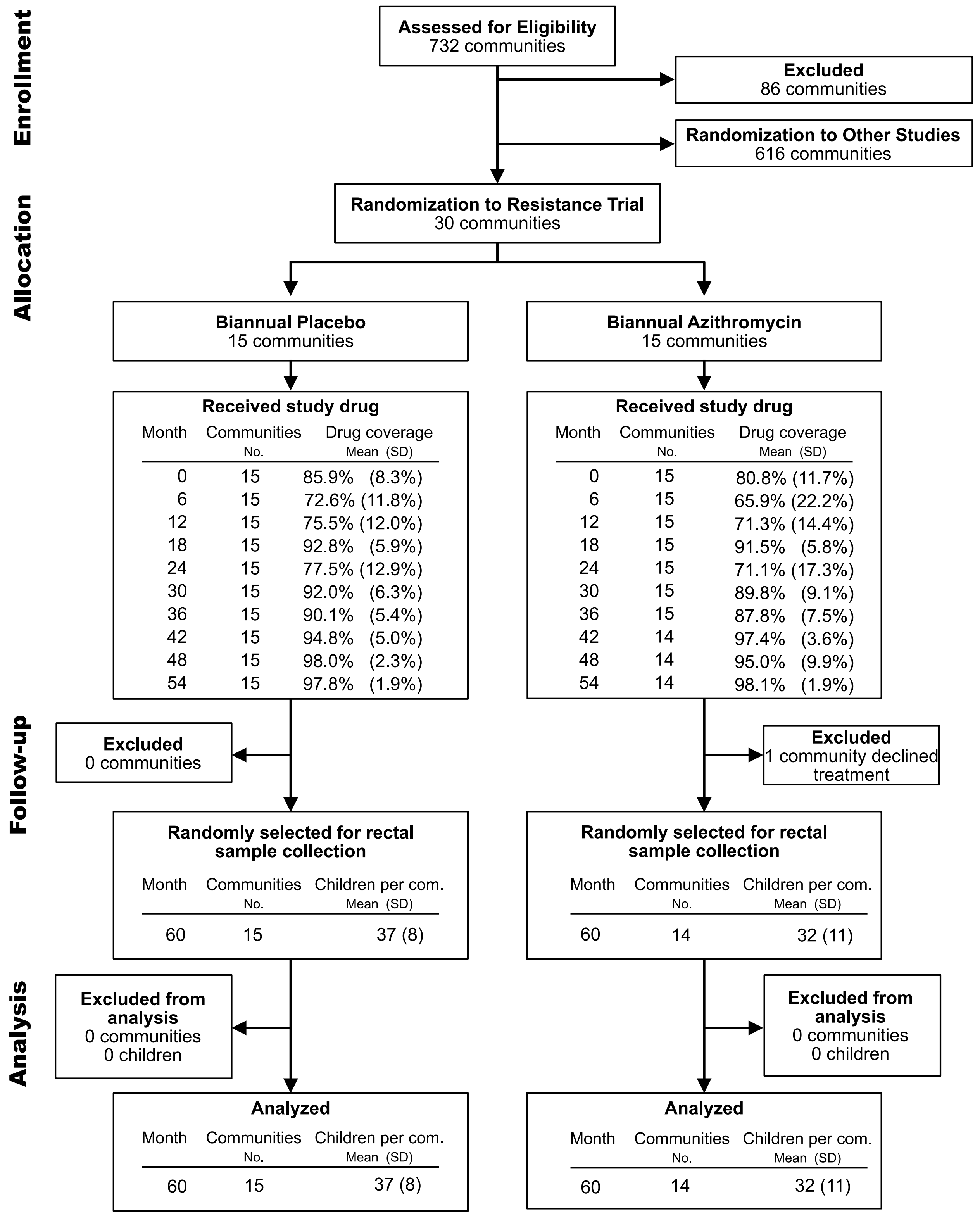

Figure S2: Normalized macrolide resistance determinants for placebo- and azithromycin-treated villages at baseline, 24, 36, 48, and 60 months. Bars indicate the mean and 95% confidence intervals. Each point represents a village.

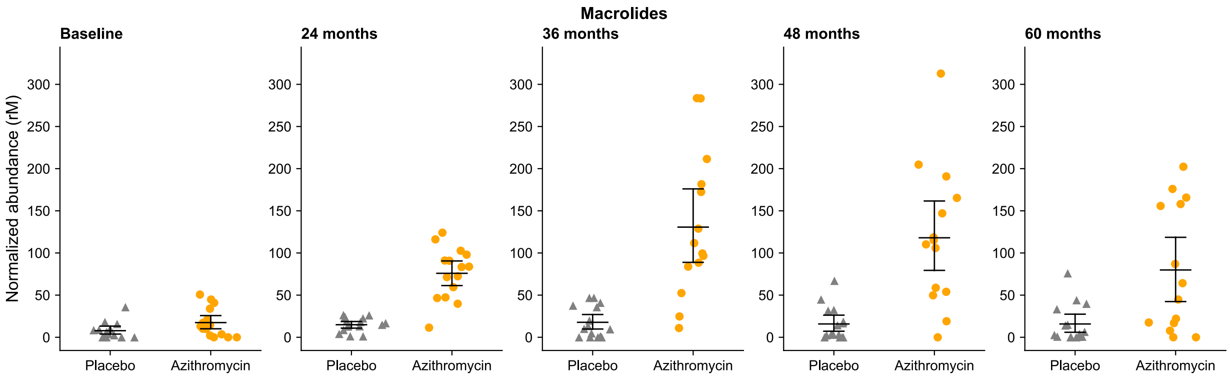

Figure S3: Normalized non-macrolide resistance determinants for placebo- and azithromycin-treated villages at 60 months. Bars indicate the mean and 95% confidence intervals. Each point represents a village.

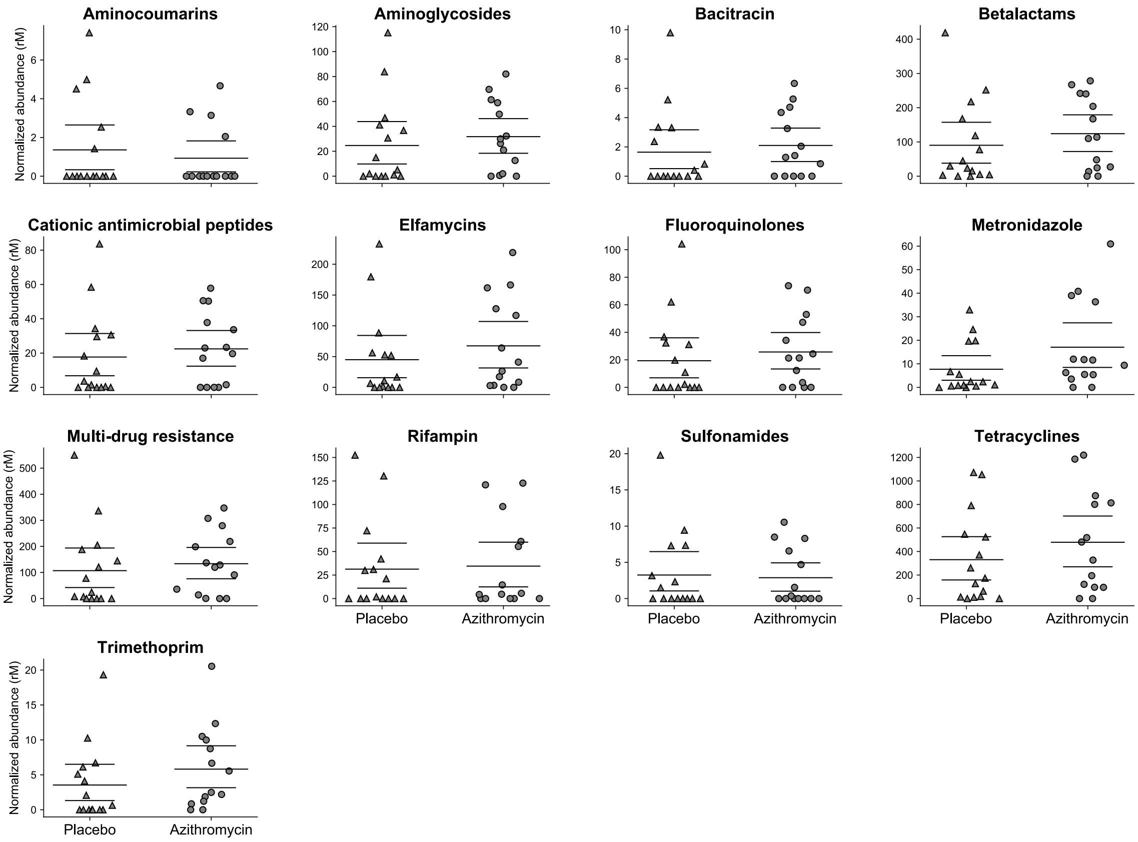

Table S1: Demographics of Analyzed Participants

	Rectal Swabs 60 months	
	Placebo	Azithromycin
Number of children	557	447
Number of communities	15	14
Mean age, months (95% CI)	32 (31 to 33)	32 (30 to 34)
Female, % (95% CI)	48 (44 to 52)	45 (39 to 50)
